# Broad Adaptive Immune Responses to *M. tuberculosis* Antigens Precede TST Conversion in Tuberculosis Exposed Household Contacts in a TB-Endemic Setting

**DOI:** 10.1371/journal.pone.0116268

**Published:** 2014-12-30

**Authors:** Ulrike K. Buchwald, Ifedayo M. O. Adetifa, Christian Bottomley, Patrick K. Owiafe, Simon Donkor, Adama L. Bojang, Jayne S. Sutherland

**Affiliations:** 1 Vaccinology Theme, Medical Research Council Unit, Banjul, The Gambia; 2 Disease Control and Elimination Theme, Medical Research Council Unit, Banjul, The Gambia; 3 Medical Research Council Tropical Epidemiology Group, London School of Hygiene and Tropical Medicine, London, United Kingdom; University of Cape Town, South Africa

## Abstract

**Background:**

The identification of *Mycobacterium^-^tuberculosis* (Mtb) infected individuals remains a challenge due to an insufficient understanding of immune responses detected with the current diagnostic tests for latent tuberculosis i.e. the tuberculin skin test (TST) or IFN–γ release assays (IGRAs) and an inability to distinguish infection stages with current immunologic assays. Further classification based on markers other than IFN–γ may help to define markers of early Mtb infection.

**Methods:**

We assessed the TST status of Mtb^-^exposed household contacts at baseline and at 6 months. Contacts were classified into those with initial positive TST (TST^+^); those with baseline negative TST but TST conversion at 6 months (TST converters, TSTC) and those with persistently negative TST (PTST^−^). We assessed their short^-^ and long^-^term immune responses to PPD and ESAT–6/CFP–10 (EC) via IFN–γ ELISPOT and a multiplex cytokine array in relation to TST status and compared them to those of TB cases to identify immune profiles associated with a spectrum of infection stages.

**Results:**

After 1 and 6 days stimulation with EC, 12 cytokines (IFN–γ, IL–2, IP–10, TNF–α, IL–13, IL–17, IL–10, GMCSF, MIP–1β, MCP–3, IL–2RA and IL–1A) were not different in TSTC compared to TST^+^ suggesting that robust adaptive Mtb^-^specific immune responses precede TST conversion. Stratifying contacts by baseline IFN–γ ELISPOT to EC in combination with TST results revealed that IP–10 and IL–17 were highest in the group of TST converters with positive baseline ELISPOT, suggesting they might be markers for recent infection.

**Conclusion:**

We describe a detailed analysis of Mtb^-^specific biomarker profiles in exposed household contacts in a TB endemic area that provides insights into the dynamic immune responses to Mtb infection and may help to identify biomarkers for ‘at^-^risk’ populations beyond TST and IGRA.

## Introduction

About one–third of the world's population harbors a latent infection with *Mycobacterium tuberculosis* (Mtb), the causative agent of tuberculosis (TB), creating a reservoir for the development of active disease and continued transmission. Mtb infection is characterized by a complex interplay of bacterial metabolic and replicative stages and host immune responses [1]. Detection of T–cell sensitization to Mtb antigens has traditionally been used for diagnostic purposes but has also been associated with the quest to understand the dynamics of host immunity and protection, which would greatly facilitate development of optimal therapeutics and vaccines. Current immune based diagnostic methods include the tuberculin skin test (TST) and IFN–γ release assays (IGRA) utilizing overnight stimulation with dominant Mtb antigens such as ESAT–6/CFP–10 (EC). However, there is a significant discordancy between TST and IGRA, which is incompletely understood and might be associated with host genetic and/or environmental factors. Both assays are also currently not able to accurately discriminate different stages of Mtb infection. Recent studies have addressed whether quantitative or qualitative differences in immune profiles, gene expression pattern and functional T–cell signatures between LTBI and TB cases can be utilized for diagnostic purposes [2]–[8].

In this regard, has become clear that IFN–γ alone is not definitively a marker for a protective immune response to Mtb [9], [10] and strong IFN–γ and TNF–α responses might even be associated with immune pathology [4]. Conversely, IL–2 secreting central memory cells are associated with latent TB infection (LTBI) and appear in response to successful TB treatment [11]. The presence of T cells producing IL–17 during a secondary immune response is thought to be important for protection against active TB [12], while innate immunity is essential for protective immunity to Mtb infection prior to development of latency.

Few studies have looked at immune profiles in household contacts based on longitudinal TST status and have simultaneously assessed TST status and responses in IGRAs. In Uganda, no differences in innate immune responses were seen between exposed household contacts that remained TST negative for 1 year compared to those that converted their TST. However, TST converters had higher baseline IFN–γ responses to Mtb bacilli, culture filtrate protein (CFP) and antigen 85 B (Ag85B) [13]. Only IFN–γ production was assessed and thus the investigators may have missed key information provided by other cytokines (e.g. Th1, Th2, Th17). Another study in Pakistan showed no differences in the baseline levels of IFN–γ, TNF–α, IL–10 and IL–6 between TST^+^ contacts and TST converters; however, all of their initially TST negative contacts has converted by 6 months; hence no persistently TST negative contacts were studied [14].

As part of the on–going TB case^-^control studies at the MRC Unit in The Gambia [15], we analyzed global cytokine profiles using multiplex cytokine arrays across different infection stages, including TB cases and exposed household contacts to elucidate whether quantitative or qualitative differences in biomarkers exist between different infection stages and LTBI phenotypes as defined by TST and IGRA responses. We used overnight and long^-^term cultures as it has been shown in endemic countries that long–term responses can identify persons with Mtb immune responses not evident in overnight assays and help to explain discordant results of TST and IGRAs [16]–[19]. We show that quantitative differences in cytokine responses exist between infection stages and are dependent on the length of *in vitro* stimulation. Most importantly, more than one third of contacts with initially negative TST converted by 6 month and showed broad MTB specific immune responses already at enrolment compared to persistent TST^−^ contacts. These data add to the understanding of early immune responses to TB infection and to the interpretation of those immune responses for the diagnosis of latent TB and may help to identify novel biomarkers for ‘at risk’ populations.

## Materials and Methods

### Ethics

This study was approved by The Gambia Government/MRC joint Ethics Committee and the Institutional Review Board of New York University School of Medicine. Written informed consent was obtained from all participants. All data was anonymized using a unique Subject Identifier.

### Subjects

Adult TB index cases ≥18 years of age with newly diagnosed smear^-^positive pulmonary TB were recruited from major government health centers and the outpatient and TB clinics at the MRC Unit, The Gambia. All cases had sputum samples obtained for Ziehl–Neelsen smear microscopy and TB culture. Household contacts were included if they were aged ≥18 years of age, lived in the same compound as the index case for >3 months, and were not treated for TB in the past year or diagnosed with TB within 1 month of recruitment. After a blood draw, the TST was carried out with 2 TU (PPD RT23, Statens Serum Institute, Copenhagen, Denmark) using the Mantoux method. Indurations were recorded at 48–72 hours. Subjects with a positive TST (induration diameter ≥10 mm) were offered a chest X^-^ray and those with symptoms underwent a clinical assessment. Household contacts with a negative TST at enrolment were invited to repeat the test after 6 months. TST conversion was defined as a positive test (≥10 mm induration) plus an increase in induration of at least 6 mm [20]. Of initially TST^−^ contacts, only those with a repeat TST test at month 6 were enrolled in the biomarker analysis. 2 TST^−^ contacts showed a reduction of induration from 8 and 9 mm, respectively, to 0 mm at 6 month and were excluded from the analysis due to low numbers of contacts with reduction of TST size. All cases and contacts underwent testing for HIV1/2 (Hexagon HIV; Human Diagnostics GmbH, Wiesbaden, Germany). Those with a positive screen were excluded from the biomarker analysis.

### Antigens

Pooled sequential, overlapping 15^-^mer peptides, of ESAT^-^6 and CFP^-^10 proteins were used at a final concentration of 2.5 µg/ml each (Advanced Biotechnology Centre, Imperial College, UK) [21]. Purified Protein Derivative (PPD) (SSI, Copenhagen, Denmark) was used at 10 µg/ml. The polyclonal stimulator Phytohemagglutinin (PHA, Sigma^-^Aldrich, St. Louis, MO, USA) at 5 µg/ml served as a positive control and culture medium (RPMI 1640 (Sigma Aldrich) containing Penicillin (10 IU/mL), Streptomycin (10 µg/mL), L–Glutamine (2 mM)) served as a negative control.

### Preparation of peripheral blood mononuclear cells (PBMC)

PBMC were prepared from heparinized blood using Lymphoprep^TM^ (Axis Shield, Oslo, Norway). Red blood cells were lysed with ACK lysis buffer. All culture assays were performed on freshly isolated cells.

### *Ex vivo* IFN–γ ELISPOT

*Ex vivo* ELISPOT assays for IFN–γ were performed as previously described after stimulation with ESAT–6/CFP–10 and PPD [22]. Positive test wells were predefined as containing 30 spot–forming units (SFU)/10^6^ cells more than – and at least twice as many as – negative control wells [23]. PHA positive controls were set to 750 SFU/10^6^ cells above negative controls. Negative control wells were required to have <100 SFU/10^6^ cells.

### Cultured IFN–γ ELISPOT

A 10 day cultured ELISPOT was performed as previously described [24]. In brief, 1×10^6^ PBMCs were incubated with antigen (EC or PPD) in 500 µl culture medium with 10% human AB serum (Sigma Aldrich). On days 5 and 7, 250 µl of medium were replaced with fresh medium containing IL–2 (Chiron, Charlotte, NC, USA) in a final concentration of 10 IU/ml. On day 9 the cells were washed three times, re^-^suspended in 1 ml medium, and rested overnight. On day 10, cells were re^-^stimulated as described for the *ex vivo* ELISPOT and processed on day 11. A positive cultured IFN–γ ELISPOT was defined as having >300 SFU/10^6^ cells above the negative control with at least double the number of the negative control [24]. The positive PHA controls had to have >1000 SFU/10^6^ cells.

### Cell culture for cytokine assays

2×10^5^ PBMC/well were placed in culture medium containing 5% AB serum for overnight stimulation and 10% AB serum for 6 day stimulation; then they were cultured in 96–well U-bottom tissue culture plates (BD, Franklin Lakes, NJ, USA) in the presence of EC or medium control. 150 µl of cell^-^free supernatant was harvested from each well and duplicates were combined in a new plate and stored at −20°C until required.

### Multiplex bead analysis for cytokine/chemokine production

A 17–plex MILLIPLEX^TM^ MAP Kit (Millipore Corp, Billerica, MA, USA) was used to measure levels of IFN–γ, IL–2, TNF–α, IL–12p70, GMCSF, IL–4, IL–13, IL–10, IL–2ra, IL–1a, IL–17, MCP–3, MIP–1β, MIP–1α, IL–12p70, CD40L and VEGF in supernatants obtained on days 1 and 6 according to the manufacturer's instructions [25]. The data for MIP–1α, IL–4, IL–12p70, CD40L and VEGF were not included in our analysis as they were above (MIP–1α) or below (all others) the limits of standard curve range for the majority of samples in the study population. The inter–assay coefficient of variation for the quality controls ranged from 11–23%, similar to described ranges [26]. Cytokine/chemokine levels are expressed in pg/ml. All laboratory assays were done blinded to the subjects' group.

### Statistical analysis

Household contacts were categorized according to their TST status at screening and 6 month follow up: TST positive at screening (TST^+^), TST negative at screening and positive at 6 month (TST converters = TSTC) and TST negative at both time points (persistently TST^−^ = PTST^−^). Antigen^-^specific cytokine/chemokine levels were obtained by subtracting the levels of the un–stimulated well (negative control) from the antigen–stimulated well. For each analyte, negative and zero values were set to half the lower limit of detection. To account for cytokine variance, the geometric mean was calculated for each group (TB cases, TST^+^, PTST^−^, TSTC). For the cytokine/chemokine analyses, the ratio of the geometric means was calculated between groups. P–values for comparisons between groups were calculated using the Z–test to allow for unequal variances. Comparisons were also adjusted for household, age and sex. All listed p–values in the text are from the adjusted analysis; the unadjusted analysis for the multiplex assay can be found in the supplementary tables. The demographic characteristics of the groups were compared using Fisher's exact test (categorical variables) or Kruskal–Wallis test (continuous variables). A p–value ≤0.05 was considered statistically significant. Statistical analyses were performed using Stata/IC software version 11.0 (StataCorp, College Station, TX, USA) and GraphPad Prism software version 6.0 (Software Mackiev, USA).

## Results

### Study subjects

23 HIV^-^negative TB patients and 129 HIV–negative household contacts were included in our final biomarker analysis. 64 household contacts were TST positive (TST^+^) at the time of enrolment. Of the 65 TST negative (TST^−^) contacts, 26 (40%) showed TST conversion (converters = TSTC) at month 6 while 39 (60%) remained TST negative (persistently TST^−^ = PTST^−^). There was no difference in the presence of a BCG scar, BMI index or hematology parameters based on TST status (Table 1, data for BMI and hematology not shown). However, contacts with initially positive TST were more likely to sleep in the same bedroom of the index case than PTST^−^ (Odds Ratio (OR) 4.3, [95% Confidence Interval (CI) 1.11–24.57], p = 0.02; Table 1). No statistical significant differences were seen in the smear grade of index cases between groups, the time to culture positivity or the prevalence of *M. tuberculosis* versus *M. africanum* (Table 1).

**Table 1:** Demographic, microbiologic and Mtb exposure characteristics of study participants.

	Cases (n = 23)	TST^+^ (n = 64)	TSTC (n = 26)	PTST^−^ (n = 39)	p–value
Age, median (range)	31 (27–35)	29 (20–38)	29 (23–49)	28 (20–45)	ns
Male gender, n (%)	16 (70)	32 (50)	13 (50)	16 (41)	ns
Presence of a BCG scar, n (%)	9/20 (45)	35/63 (56)	16/26 (62)	19/39 (49)	ns
Baseline TST size, median (min., max, IQR)	0 (0,16,0)	14 (10,28,9)	0 (0,5,0)	0 (0,5,0)	p<0.001
6 month TST size, median (min., max; IQR)	na	na	18 (10,25,6)	0 (0,5,0)	p<0.001
**Sleeping Proximity to Index Case**					
Same bedroom, n (%)		17 (27)	3 (11)	3 (8)	
Different bedroom, n (%)		29 (45)	15 (58)	16 (41)	
Different house, n (%)		18 (28)	8 (31)	20 (51)	p = 0.041*
**Smear Grade of Index Case**					
Grade 1, n (%)	2 (9)	9 (14)	1 (4)	8 (21)	
Grade 2, n (%)	9 (39)	27 (42)	12 (46)	15 (38)	
Grade 3, n (%)	12 (52)	28 (44)	13 (50)	16 (41)	ns
**Spoligotype of Index Case where known**	**n = 21**	**n = 58**	**n = 24**	**n = 34**	
*M. tuberculosis*, n (%)	11 (52)	35 (60)	19 (79)	27 (79)	
*M. africanum*, n (%)	10 (48)	23 (40)	5 (21)	7 (21)	ns
**Days to Positivity of Index Case Cultures**					
Days, median (IQR)	9 (17)	12 (17)	6.5 (10)	6 (18)	ns

* Fisher's exact test: p = 0.017 for proximity between TST^+^ and PTST^−^. ns = not significant; TST^+^ = TST positive; TSTC = TST converters; PTST^−^ = persistently TST negative; na = not assessed; IQR = interquartile range.

### IFN–γ ELISPOT following ESAT–6/CFP–10 and PPD stimulation further defines TST phenotypes in household contacts

**Figure 1:**
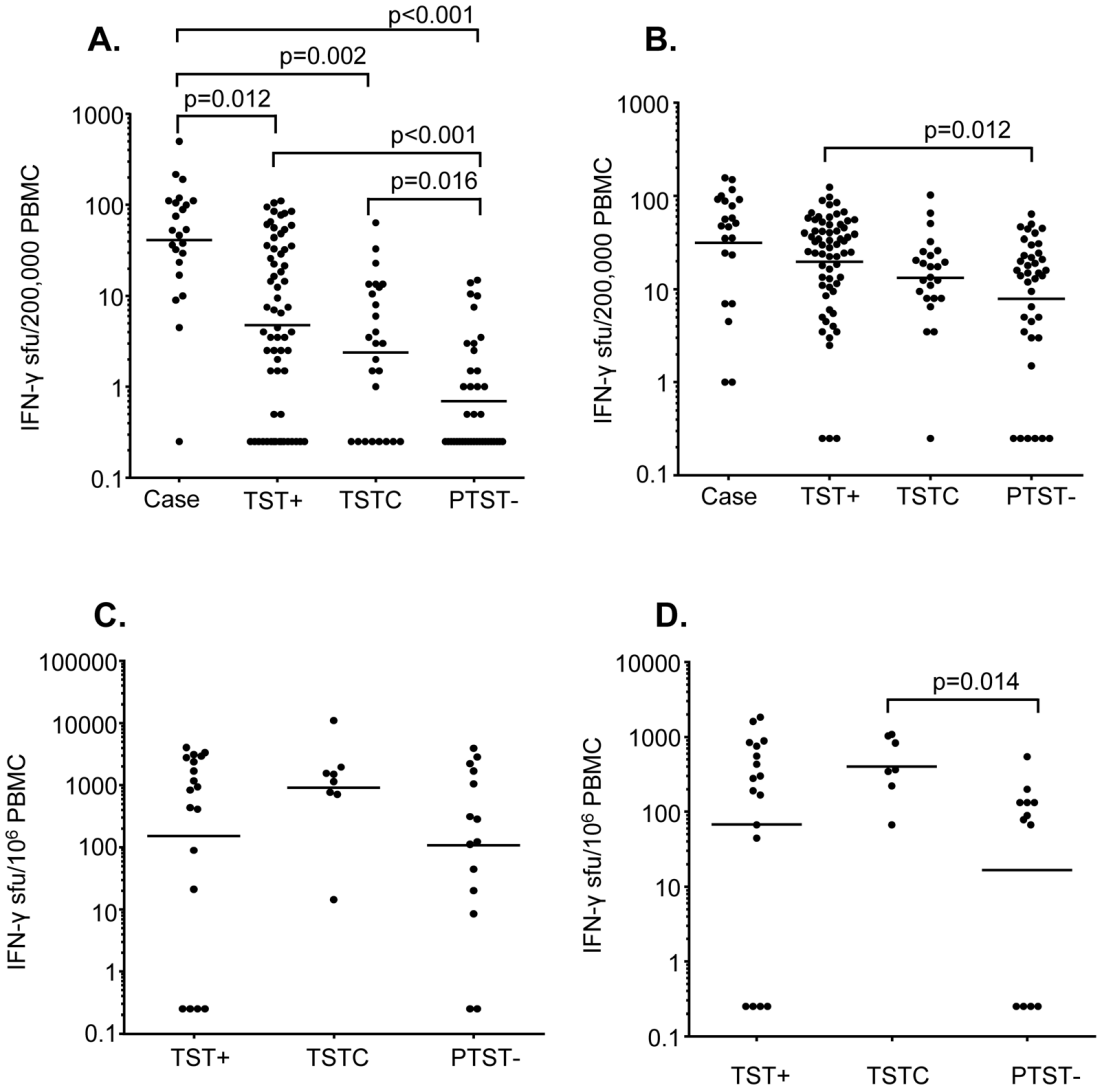
IFN–γ ELISPOT in cases and household contacts in response to stimulation with ESAT–6/CFP–10 (EC) or Purified Protein Derivative (PPD), at enrolment. Peripheral blood mononuclear cells (PBMC) from TB cases and contacts were stimulated overnight (A and B) or for 6 days (C and D) with EC (A and C) or PPD (B and D) and an IFN–γ ELISPOT was performed. Horizontal lines represent the geometric mean of the spot forming units (SFU). Contacts were categorized according to TST scores at baseline and 6 months: TST^+^ = TST positive: TST≥10 mm at baseline; TSTC = TST converters: TST<10 mm at baseline and TST≥10 mm plus an increase in induration of at least 6 mm by 6 month; PTST^−^: persistently TST negative: TST<10 mm at both time^-^points. P^-^values represent comparisons adjusted for household, sex and gender.

All subjects had an overnight IFN^-^γ ELISPOT with EC and PPD stimulation performed at baseline. After stimulation with EC, 32/64 (50%) of TST^+^ subjects and 14/65 (22%) of TST^−^ contacts were ELISPOT^+^ (ECS^+^) (OR 3.64 [CI 1.59–8.51], p<0.001). In relation to the 6 month TST status, the baseline EC ELISPOT was positive in 9/26 (35%) of TSTC and 5/39 (13%) of PTST^−^ (OR 3.6 [CI 0.9–15.6], p = 0.0625). TST^+^ and TSTC subjects had significantly higher spot forming units (SFU) counts following EC stimulation than PTST^−^ subjects (TST^+^: Geometric Mean (GM) [95% confidence interval (CI)]: 24 [CI 14–41] SFU/10^6^ cells; TSTC: 12 [CI 6–25], PTST^−^: 3.5 [CI 2–5], TST^+^ vs PTST^−^: p<0.001, TSTC vs PTST^−^: p = 0.016 (all analyses adjusted for household, sex, gender) while there was no difference between TST^+^ contacts and TSTC (Fig. 1A). 21/23 (91%) of TB cases had a positive response and the GM SFU counts were significantly higher than for TST^+^, TSTC and PTST^−^ (p = 0.012, p = 0.002 and p<0.001 respectively; Fig. 1A).

After overnight stimulation with PPD, 86% of TB cases, 86% of TST^+^, 88% of TSTC and 72% of PTST^−^ contacts had a positive ELISPOT. PTST^−^ had a significantly lower GM SFU count compared to TST^+^ contacts (TST^+^: 99 [CI 70–138] SFU/10^6^ cells, PTST^−^: 39 [CI 23–69], p = 0.012). PTST^−^ and TSTC had lower SFU counts than TB cases but the difference was not significant in the adjusted analysis (Fig. 1B).

A cultured ELISPOT utilizing both antigens was performed for the last 40 household contacts entering the study: 18 TST^+^, 8 TSTC and 14 PTST^−^. In response to EC, TSTC had a higher GM SFU count than both TST^+^ and PTST^−^ but the difference did not reach statistical significance after adjusting for age, sex and household (Fig. 1C). However, TSTC had a significantly higher response to PPD compared to PTST^−^ (TSTC: GM 352 [CI 152–816], PTST^−^: GM 43 [CI 14–134], p = 0.014; Fig. 1D).

Interestingly, TST^+^ contacts with a positive overnight IFN–γ ELISPOT had a significantly larger TST size at enrolment than TST^+^ contacts with a negative ELISPOT (Median [IQR]: 18 [10] mm vs 10 [6] mm, p = 0.002). For the TST converters, median TST size at 6 months was 18 [6] mm, which was not statistically different from the median TST size for TST^+^ contact at enrolment (14 [8] mm). No difference in the TST sizes were seen between TSTC and PTST^−^ at the baseline measurement and no difference in the TST size at 6 months between TSTC with positive versus negative baseline IFN–γ ELISPOT, respectively.

### Cytokine/chemokine signatures in PTST^−^ are different from TSTC, TST^+^ and TB cases

**Figure 2:**
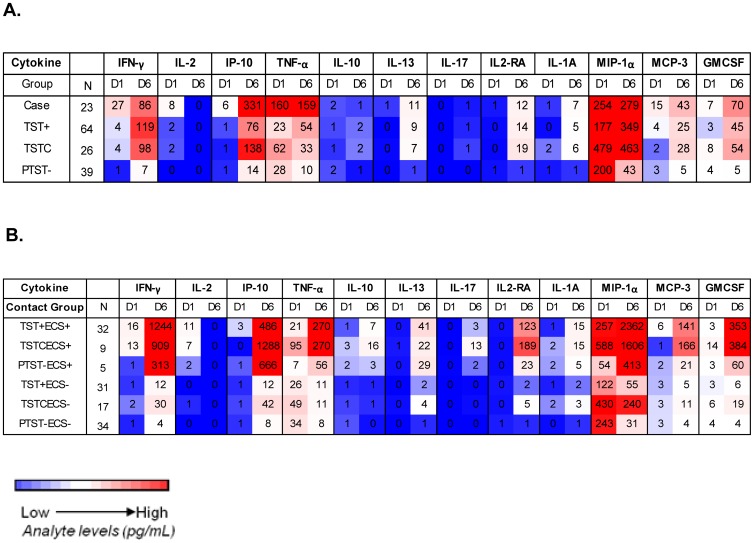
Cytokine/chemokine responses in TB cases and household contacts stratified by TST status after stimulation with ESAT^-^6/CFP^-^10 (EC) at enrolment. Following overnight or 6 day culture of peripheral blood mononuclear cells with EC, supernatants were collected and multiplex cytokine assays performed. Geometric mean levels are shown (pg/ml) A: Heat map showing geometric mean levels of different analytes after overnight (D1) versus 6 day (D6) culture in cases and contacts based on TST phenotype. The highest geometric means are shaded in red and the lowest in blue. Contacts were categorized according to TST scores at baseline and 6 months: TST^+^ = TST positive: TST≥10 mm at baseline; TSTC = TST converters: TST<10 mm at baseline and TST≥10 mm plus an increase in induration of at least 6 mm by 6 month; PTST^−^: persistently TST negative: TST<10 mm at both time–points. B: Heat map showing geometric mean levels of different analytes after overnight (D1) and 6 day (D6) culture in contacts grouped according to TST status and baseline EC IFN^−^γ ELISPOT (ECS) results. The highest geometric means are shaded in red and the lowest in blue.

Cytokine levels measured on day 1 and day 6 of EC stimulation were analyzed for TB cases, TST^+^, TSTC and PTST^−^. The highest responses were seen for IFN–γ, IP–10, TNF–α, MIP–1β and GMCSF for all groups (Fig. 2A; see S1 and S2 Tables for details and the unadjusted analysis). Most day 6 cytokines levels were higher than those measured on day 1, except for MIP–1β, TNF–α and IL–2. Of note, IL–2 levels were very low on day 6 in all groups (Fig. 2A). IFN–γ, IL–2, MCP–3 and IL–13 were significantly higher in TB cases than in PTST^−^ when adjusted for household, age and sex (S1 Table). Cases had significantly higher GM of IFN–γ and TNF–α than TST^+^ (p<0.05) and higher IP–10 and MCP–3 than TSTC (p<0.01, S1 Table). The GM of IL–2 was significantly lower in PTST^−^ as compared to TST^+^ and TSTC and the GMs of IFN–γ and IL–13 as compared to TSTC (p<0.05). No significant differences in any cytokine were seen for TST^+^ compared to TSTC (Fig. 2A, S1 Table). After 6 days *in vitro* stimulation with EC, the GM of IP–10 and IL–13 were significantly higher in TB cases compared to PTST^−^ (p<0.05). PTST^−^ subjects also had significantly lower GM of IFN–γ, MIP–1β, IL–2RA and IL–13 levels than TST^+^ contacts (p<0.05). Compared to TSTC, PTST− contacts had lower GM of all analytes except IL–2, TNF–α, IL–10 and IL^−^1A (p<0.05 for MCP–3, IL–13, IL–17; p<0.01 for IFN–γ, IP–10, MIP–1β, IL–2RA, GMCSF). No significant differences in any cytokine were seen between TST^+^ and TSTC (Fig. 2A, S2 Table).

### Responsiveness in the ESAT–6/CFP–10 ELISPOT defines subgroups of TST converters and persistently TST negative contacts

We have previously shown that a positive ESAT–6/CFP–10 IFN–γ ELISPOT in TST^−^ contacts is a strong predictor of subsequent TST conversion [20]. In the present study, 64% of the TST negative contacts with a positive baseline EC–ELISPOT converted their TST by 6 month compared to only 33% of those that were baseline EC–ELISPOT negative (OR 3.6 [CI 0.9–15.6]; not signficant). Furthermore, TSTC showed strong IFN–γ responses to mycobacterial antigens in the long–term ELISPOT assays as compared to PTST^−^ subjects. We analyzed multi^-^cytokine profiles in these subjects to determine further differences in innate and adaptive host markers based on TST and EC status. Six groups were defined: TST^+^ECS^+^ (n = 32), TST^+^ECS^−^ (n = 31), TSTCECS^+^ (n = 9), TSTCECS^−^ (n = 17), PTST^−^ECS^+^ (n = 5), PTST^−^ECS^−^ (n = 34) (Fig. 2B, S3 and S4 Tables for details and the unadjusted analysis). The highest cytokine responses were seen for IFN–γ, IP–10, TNF–α, MIP–1β and GMCSF, especially after 6 days stimulation. However, the analysis based on ECS status also revealed strong MCP–3 and IL–2RA responses in ECS^+^ contacts with positive baseline TST or TST conversion (Fig. 2B). After overnight stimulation, the GM of IFN–γ and IL–2 were significantly higher in TST^+^ECS^+^ and TSTCECS^+^ than in TST^+^ECS^−^, PTST^−^ECS^−^ and TSTCECS^−^ (TSTCECS^+^ vs TSTCECS^−^: all p<0.05; TST^+^ECS^+^ vs TSTCECS^−^: IFN–γ: p^-^0.023; TST^+^ECS^+^ vs TST^+^ECS^−^: IFN–γ: p = 0.003, all others: p<0.001). GM levels of IFN–γ and IL–2 in PTST^−^ECS^+^ were lower than in TST^+^ECS^+^ and TSTCECS^+^ with only the difference in IFN–γ to TSTCECS^+^ being significant in the adjusted analysis (p = 0.02). However, PTST^−^ECS^+^ had significantly higher IL^-^2 levels than TST^+^ECS^−^ and PTST^−^ECS^−^ (p≤0.002). TSTC with negative ECS had higher GM IFN–γ and IL–2 levels than TST^+^ECS^−^ and PTST^−^ECS^−^ but differences were not significant in the adjusted analysis (Fig. 2B, S3 Table).

On day 6, TST^+^ECS^+^ and TSTCECS^+^ had significantly higher GM cytokine/chemokine levels than TST^+^ECS^−^ and PTST^−^ECS^−^ and the difference was significant for all analytes except for IL^-^13 between TSTCECS^+^ to TST^+^ECS^−^ (p≤0.05 for IL–13, IL–17, IL–1A; p≤0.01 for TNF–α, IL–10, IL–13 and IL–17; p≤0.001 for all others; Fig. 2B, S4 Table). However, compared to TSTCECS^−^, only TNF–α, MCP–3 and IL–10 were significantly higher in TST^+^ECS^+^ and TSTCECS^+^ as well as IFN–γ in TST^+^ECS^+^ and IP–10, IL–17 and IL–10 in TSTCECS^+^ (p≤0.05). TSTCECS^+^ also had several times higher level of IP–10 and IL–17 compared to TST^+^ECS^+^ (IP–10: p = 0.037; IL–17: p = 0.056).

PTST^−^ECS^+^ had higher GM of IFN–γ, IP–10, MIP–1β, IL–2RA and IL–1A than TST^+^ECS^−^ (p<0.05 for IL–1A, IL–2RA and MIP–1β; p<0.001 for IFN–γ, IP^-^10) and higher IFN–γ and IP–10 than TSTCECS^−^ (IFN–γ: p = 0.034; IP–10: p = 0.001). GMs of IFN–γ, IP–10, IL–13, IL–2RA, GMCSF, MIP–1β and IL–1A were also higher in this group as compared to PTST^−^ECS^−^ (p≤0.05 for IL–1A, IL–13, MIP–1β, GMSCF, p<0.001 for IFN–γ, IP–10). Among the ECS^−^ groups, TSTCECS^−^ had significantly higher levels MIP–1β and IL–13 than PTST^−^ECS^−^ (p<0.05). Adjusting for sleeping proximity did not change which of the day 1 and day 6 comparisons were significant (data not shown).

## Discussion

To our knowledge, this is the first study to evaluate a panel of Mtb^-^specific Th1, Th2, Th17 and innate cytokine/chemokine responses in exposed contacts of TB index cases stratified by longitudinal TST results and baseline IGRA results. Importantly, we analyzed cytokine responses in both short and long^-^term cultures with Mtb^-^specific antigens, which revealed important differences between infection stages. Short^-^term cultures mainly detect circulating effector memory response, long^-^term cultures are thought to reflect central memory cells [19].

One half of exposed contacts had a positive TST at baseline and 40% of the TST negative contacts showed TST conversion by 6 months. Positive results in the overnight IFN–γ ELISPOT decreased from 50% in TST^+^ contacts and 33% in TST converters to only 13% in persistently TST negative contacts.

One of the main findings of our study is that TST converters already showed broad EC responses at recruitment with no quantitative or qualitative differences compared to TST^+^ contacts regardless of the culture time. In contrast to TB cases, TST converters and TST^+^ contacts had lower overnight responses in the EC and PPD ELISPOT assays. TSTC also had lower IP–10 and MCP–3 levels in the overnight assay while IFN–γ and TNF–α levels were lower in TST^+^ contacts than in cases. However, on day 6, no significant differences were seen in the adjusted analysis between both contact groups and cases. These differences between short and long–term assays in cases versus contacts could be due to a decreased viability and increased apoptosis of T–cells from TB patients in 6 day cultures as has been recently described [27], [28] and this was not tested at day 6 in the current study. However, they are also in line with previous findings that different cell populations are the source of the cytokines/chemokines such as effector memory cells in cases versus central memory cells in LTBI [29]–[31]. For example, Marin et al found an increase in single and double cytokine^-^producing CD4^+^ and CD8^+^ T cells in response to PPD in LTBI subjects between day 1 and day 6 and in single and triple cytokine^-^producing T cells in response to irradiated Mtb.

Contacts who did not convert their TST had significantly lower IFN–γ responses than TST converters and TST^+^ contacts in the short^-^and long–term EC and PPD ELISPOT assays, lower IFN–γ and IL–2 responses in the short term multiplex assay and lower levels of most cytokines except IL–2 and TNF–α in the long–term multiplex assay. These results are similar to findings in a Ugandan study where TSTC had higher IFN–γ responses to *Mycobacterium tuberculosis* (Mtb), Culture Filtrate (CF) and Antigen 85B (Ag85B) than PTST^−^ [13]. However, in that study, the baseline IFN–γ responses to all 3 antigens were still significantly lower in TSTC compared to TST^+^ contacts and only reached similar levels by 3 months (Ag85B) or even 9–11 months (Mtb, CF). While we were not able to study longitudinal cytokine responses, our TSTC had strong immune responses already at enrolment with no differences to TST^+^ contacts. This difference might be due to variances in the length and strength of the exposure to TB cases before enrolment, the different Mtb antigens used for stimulation and/or methodological differences. Another HHC study in Pakistan found no significant difference in baseline IFN–γ, TNF–α, IL–6 or IL–10 to Culture Filtrate (CF) between TST^+^ and TSTC; however the latter had a steeper increase of IFN–γ, IL–6 and IL–16 over the 24 months of follow^-^up compared to our study [14]. Together these results emphasize that a robust adaptive Mtb specific immune responses precede TST conversion.

In a previous study in The Gambia, we have shown that a positive recruitment ELISPOT to EC is a strong predictor of subsequent TST conversion [20]. Indeed, TST^+^ contacts and TST converters with a positive EC ELISPOT showed high positive IFN–γ responses after EC stimulation as expected, but also of all the other cytokines studied. The only difference between TST^+^ECS^+^ and TSTCECS^+^ were higher IP–10 and IL–17 levels in the latter. IL–17 is a pro–inflammatory cytokine produced by antigen specific CD4 cells which can enhance a Th1 memory response but potentially also contribute to lung inflammation by attracting neutrophils to lung tissue [32], [33]. A recent study showed that 50% of individuals with LTBI have Mtb specific IL–17 responses while these responses are absent in TB patients [34]. Similarly, Hur et al found lower IL–17 levels in Malawian TB patients than in their spouses [35]. In our study, IL–17 responses were generally low except in the group of TSTCECS^+^, suggesting that it corresponds to an early immune^-^phenotype after recent infection.

Eight percent of contacts with an initial negative TST did not convert the TST at 6 month despite a positive EC ELISPOT at baseline. Quantitative biomarker responses to EC in this group were lower compared to TST^+^ECS^+^ and TSTCECS^+^ but the differences were not significant when adjusted for sex, age and household. Furthermore, all tested cytokines were expressed in this group, including IL–10, IL–17 and IL–13. Therefore, qualitative immune differences measured in the peripheral blood in these contacts do not seem to explain the lack of TST conversion. Quantitative differences could contribute, especially if maintained over time, and could be related to exposure characteristics or the virulence of the infecting organism. However, adjusting for sleeping proximity did not alter the significant results of our analysis. It is more likely that host factors and genetic determinants contribute to the absence of a DTH response to PPD in the skin despite positive acquired T–cell responses in the blood. A limitation of this study was the absence of a follow^-^up ELISPOT at 6 months, thus we do not know whether the PTST^−^ECS^+^ contacts still had a positive ELISPOT by 6 months or whether they underwent ELISPOT reversion. This is important to determine in future studies since it has been shown that patients with initially negative TST but positive IGRA response are more likely to undergo IGRA reversion over time [22], [36], [37]. In the overnight EC ELISPOT, PTST^−^ECS^+^ contacts had lower SFU counts than TST^+^ECS^+^ and TSTCECS^+^. Nevertheless, despite lack of TST conversion, cytokine levels, particularly IP10, follwoing EC stimulation were higher than those of TSTCECS^−^, further supporting the importance of host factors in Mtb immune responses [38]. Studies in The Gambia and Uganda have shown that acquired immune responses to secreted Mtb antigens such as ESAT–6 and Ag85 but also the DTH response to PPD are influenced by genetic variance [39], [40] and this should also be addressed in future studies.

Interestingly, TST^+^ECS^−^ contacts had minimal responses to EC across the panel of cytokines/chemokines evaluated. Furthermore, their cytokines levels were not different from ECS^−^ contacts that remained TST negative. In addition, the TST^+^ECS^−^ subjects had a significantly smaller TST size than TST^+^ECS^+^ contacts and lower PPD responses in the ELISPOT, confirming our previous results [21]. The discrepancy between TST and IGRA in these contacts could be related to the TST response being positive due to BCG vaccination, environmental exposure to non–tuberculous mycobacteria (NTM) or a remote Mtb infection with loss of ESAT–6/CFP–10 responsiveness [41], [42]. However, some individuals may lack the immunological repertoire to respond to EC, thus limiting the sensitivity of the assay. In that context it is noteworthy that TST converters had equal TST sizes at 6 months regardless of whether the baseline EC ELISPOT was negative or positive. Limitations of the current IGRAs based on the sole measurement of IFN–γ are evident in the fact that contacts with a negative baseline TST and EC ELISPOT but TST conversion at 6 month, had significantly higher levels of MIP–1β and IL–13 than EC ELISPOT non^-^responders who remained TST negative. These cytokine responses are likely reflecting an early immune response to Mtb exposure/infection, before the IFN–γ response in the EC–ELISPOT or the PPD–DTH response can be detected. The time to TST conversion after known Mtb exposure is 3–7 weeks but might be shorter for *ex vivo* cytokine assays [20]. In contrast, no difference was seen in the cytokine profiles between TST^+^ECS^−^ and PTST^−^ECS^−^. These results emphasize that extended assays and biomarkers other than IFN–γ can increase the sensitivity of blood based tests for the diagnosis of LTBI and help to characterize the spectrum of TB infection stages. It is likely that PTST^−^ECS are truly uninfected. However, it is not possible to exclude the possibility that their innate immune responses cleared the infection before an acquired response was elicited or that their acquired immune response does not include responsiveness to PPD or EC.

The two main limitations to our study were a) a lack of follow^-^up analysis of immunological responses, particularly in regards to potential EC reversion and b) analysis of cytokine profiles at a single cell level which have been shown to help to elucidate functional signatures correlating with infection stages or protection [7]. A recent review by Prezzemolo et al summarizes current knowledge of CD4^+^ and C8^+^ cell functional signatures in tuberculosis [7]. However, our goal was to elucidate discordant results between TST and IGRAs whereas most previous studies use a single assay to define LTBI. Hence, phenotypic and functional profiling by flow cytometry should be evaluated in future studies.

In summary, we present detailed biomarker profiles of different TST/IGRA phenotypes in a TB endemic region that adds to our understanding of Mtb immune responses in TST converters, persistent negative tuberculin reactors and contacts with discordant TST and IGRA result. We show that strong immune responses to EC and PPD are detectable in many contacts prior to TST conversion. Our results suggest a role for quantitative immune responses in defining infection stages. In line with previous reports, we detected additional biomarker responses to Mtb antigens in contacts in long^-^term assays that would have been missed in overnight assays and that help to explain some of the discrepant results observed between TST and IGRAs. However, our results also suggest that the observed variability and discordance of *in vitro* assays versus dermal DTH responses cannot be fully eliminated by studying cytokines other than IFN^-^γ or using long^-^term stimulation assays [43]. A better understanding of the contribution of host genetics to anti^-^mycobacterial immune responses might help to find improved markers for latent TB in different contact groups. Furthermore, it is imperative to understand whether and how the described phenotypes differ in their clinical course and risk for active TB.

## Supporting Information

S1 Table **Cytokine/chemokine responses of cases and contacts after 1 day stimulation with ESAT–6/CFP–10 (EC).** The geometric mean (GM) levels are shown in pg/ml and the ratio of the geometric mean levels is compared to TST^+^ contacts. P–values are shown for the unadjusted analysis and after adjustment for household, sex and age. ns = not significant = p>0.05. TST^+^ = TST positive at baseline; TSTC = TST converters; PTST^−^ = persistently TST negative.
(DOCX)

S2 Table **Cytokine/chemokine responses of cases and contacts after 6 day stimulation with ESAT–6/CFP–10.** The geometric mean (GM) levels are shown in pg/ml and the ratio of the geometric mean levels is compared to TST^+^ contacts. P–values are shown for the unadjusted analysis and after adjustment for household, sex and age. ns = not significant. TST^+^ = TST positive at baseline; TSTC = TST converters; PTST^−^ = persistently TST negative.
(DOCX)

S3 Table **Cytokine/chemokine responses of contacts stratified by TST and ESAT–6/CFP–10 ELISPOT (ECS) results after 1 day stimulation with ESAT–6/CFP-10.** The geometric mean (GM) levels are shown in pg/ml and the ratio of the geometric mean levels is compared to TST^+^ECS^−^ contacts. P^-^values are shown for the unadjusted analysis and after adjustment for household, sex and age. ns = not significant. ECS^+^ = positive EC ELISPOT; ECS^−^ = negative EC ELISPOT; TST^+^ = TST positive at baseline; TSTC = TST converters; PTST^−^ = persistently TST negative.
(DOCX)

S4 Table **Cytokine/chemokine responses of contacts stratified by TST and ESAT^-^6/CFP^-^10 ELISPOT (ECS) results after 6 day stimulation with ESAT–6/CFP–10.** The geometric mean (GM) levels are shown in pg/ml and the ratio of the geometric mean levels is compared to TST^+^ECS^−^ contacts. P–values are shown for the unadjusted analysis and after adjustment for household, sex and age. ns = not significant; ECS^+^ = positive EC ELISPOT; ECS^−^ = negative EC ELISPOT; TST^+^ = TST positive at baseline; TSTC = TST converters; PTST^−^ = persistently TST negative.
(DOCX)

## References

[1] Barry CE, Boshoff HI, Dartois V, Dick T, Ehrt S, et al (2009) The spectrum of latent tuberculosis: rethinking the biology and intervention strategies. Nat Rev Microbiol 7:845–855. 19855401 10.1038/nrmicro2236 PMC4144869

[2] Goletti D, Raja A, Ahamed Kabeer BS, Rodrigues C, Sodha A, et al (2010) IFN−gamma, but not IP–10, MCP–2 or IL–2 response to RD1 selected peptides associates to active tuberculosis. J Infect 61:133–143. 20470822 10.1016/j.jinf.2010.05.002

[3] Sutherland JS, de Jong BC, Jeffries DJ, Adetifa IM, Ota MO (2010) Production of TNF–alpha, IL–12(p40) and IL–17 can discriminate between active TB disease and latent infection in a West African cohort. PLoS One 5:e12365. 20811496 10.1371/journal.pone.0012365 PMC2927558

[4] Sutherland JS, Adetifa IM, Hill PC, Adegbola RA, Ota MO (2009) Pattern and diversity of cytokine production differentiates between Mycobacterium tuberculosis infection and disease. Eur J Immunol 39:723–729. 19224636 10.1002/eji.200838693

[5] Ruhwald M, Bjerregaard–Andersen M, Rabna P, Eugen–Olsen J, Ravn P (2009) IP–10, MCP–1, MCP–2, MCP–3, and IL–1RA hold promise as biomarkers for infection with *M. Tuberculosis* in a whole blood based T–cell assay. BMC Res Notes 2:19. 19193208 10.1186/1756-0500-2-19 PMC2660909

[6] Mihret A, Loxton AG, Bekele Y, Kaufmann SH, Kidd M, et al (2014) Combination of gene expression patterns in whole blood discriminate between tuberculosis infection states. BMC Infect Dis 14:257. 24885723 10.1186/1471-2334-14-257 PMC4041060

[7] Prezzemolo T, Guggino G, La Manna MP, Di Liberto D, Dieli F, et al (2014) Functional ignatures of Human CD4 and CD8 T Cell Responses to Mycobacterium tuberculosis. Front Immunol 5:180. 24795723 10.3389/fimmu.2014.00180 PMC4001014

[8] Jacobsen M, Repsilber D, Gutschmidt A, Neher A, Feldmann K, et al (2007) Candidate biomarkers for discrimination between infection and disease caused by Mycobacterium tuberculosis. J Mol Med (Berl) 85:613–621. 17318616 10.1007/s00109-007-0157-6

[9] Goldsack L, Kirman JR (2007) Half–truths and selective memory: Interferon–gamma, CD4(+) T cells and protective memory against tuberculosis. Tuberculosis (Edinb) 87:465–473. 17719276 10.1016/j.tube.2007.07.001

[10] Nunes–Alves C, Booty MG, Carpenter SM, Jayaraman P, Rothchild AC, et al (2014) In search of a new paradigm for protective immunity to TB. Nat Rev Microbiol 12:289–299. 24590243 10.1038/nrmicro3230 PMC4085047

[11] Biselli R, Mariotti S, Sargentini V, Sauzullo I, Lastilla M, et al (2009) Detection of interleukin-2 in addition to interferon-gamma discriminates active tuberculosis patients, latently infected individuals, and controls. Clin Microbiol Infect 10.1111/j.1469-0691.2009.03104.x 19886902

[12] Dheda K, Chang JS, Lala S, Huggett JF, Zumla A, et al (2008) Gene expression of IL17 and IL–23 in the lungs of patients with active tuberculosis. Thorax 63:566–568. 10.1136/thx.2007.092205 18511642

[13] Mahan CS, Zalwango S, Thiel BA, Malone LL, Chervenak KA, et al (2012) Innate and adaptive immune responses during acute M. tuberculosis infection in adult household contacts in Kampala, Uganda. Am J Trop Med Hyg 86:690–697. 22492155 10.4269/ajtmh.2012.11-0553 PMC3403758

[14] Hussain R, Talat N, Shahid F, Dawood G (2009) Biomarker changes associated with Tuberculin Skin Test (TST) conversion: a two-year longitudinal follow-up study in exposed household contacts. PLoS One 4:e7444. 19826490 10.1371/journal.pone.0007444 PMC2758599

[15] Hill PC, Ota MO (2010) Tuberculosis case-contact research in endemic tropical settings: design, conduct, and relevance to other infectious diseases. Lancet Infect Dis 10:723–732. 20883968 10.1016/S1473-3099(10)70164-X

[16] Cehovin A, Cliff JM, Hill PC, Brookes RH, Dockrell HM (2007) Extended culture enhances sensitivity of a gamma interferon assay for latent Mycobacterium tuberculosis infection. Clin Vaccine Immunol 14:796–798. 17460111 10.1128/CVI.00093-07 PMC1951098

[17] Butera O, Chiacchio T, Carrara S, Casetti R, Vanini V, et al (2009) New tools for detecting latent tuberculosis infection: evaluation of RD1-specific long-term response. BMC Infect Dis 9:182. 19930588 10.1186/1471-2334-9-182 PMC2784468

[18] Leyten EM, Arend SM, Prins C, Cobelens FG, Ottenhoff TH, et al (2007) Discrepancy between Mycobacterium tuberculosis-specific gamma interferon release assays using short and prolonged in vitro incubation. Clin Vaccine Immunol 14:880–885. 17507543 10.1128/CVI.00132-07 PMC1951056

[19] Goletti D, Butera O, Bizzoni F, Casetti R, Girardi E, et al (2006) Region of difference 1 antigen-specific CD4+ memory T cells correlate with a favorable outcome of tuberculosis. J Infect Dis 194:984–992. 16960787 10.1086/507427

[20] Hill PC, Brookes RH, Fox A, Jackson-Sillah D, Jeffries DJ, et al (2007) Longitudinal assessment of an ELISPOT test for Mycobacterium tuberculosis infection. PLoS Med 4:e192. 17564487 10.1371/journal.pmed.0040192 PMC1891317

[21] Hill PC, Fox A, Jeffries DJ, Jackson–Sillah D, Lugos MD, et al (2005) Quantitative T cell assay reflects infectious load of Mycobacterium tuberculosis in an endemic case contact model. Clin Infect Dis 40:273–278. 15655747 10.1086/427030

[22] Hill PC, Brookes RH, Fox A, Fielding K, Jeffries DJ, et al (2004) Large-scale evaluation of enzyme-linked immunospot assay and skin test for diagnosis of Mycobacterium tuberculosis infection against a gradient of exposure in The Gambia. Clin Infect Dis 38:966–973. 15034828 10.1086/382362

[23] Oxford Immunotec T–Spot.TB. Available: http://oxfordimmunotec.com/international/products-solutions/t-spot-tb/. Accessed 2014 Dec 10.

[24] Winstone N, Guimaraes–Walker A, Roberts J, Brown D, Loach V, et al (2009) Increased detection of proliferating, polyfunctional, HIV–1-specific T cells in DNA−modified vaccinia virus Ankara-vaccinated human volunteers by cultured IFN–gamma ELISPOT assay. Eur J Immunol 39:975–985. 19266489 10.1002/eji.200839167

[25] Millipore. Available: http://www.emdmillipore.com/INTL/en/product/,MM_NF-HCYTOMAG-60K#anchor_PR. Accessed 2014 Dec 10.

[26] Wong HL, Pfeiffer RM, Fears TR, Vermeulen R, Ji S, et al (2008) Reproducibility and correlations of multiplex cytokine levels in asymptomatic persons. Cancer Epidemiol Biomarkers Prev 17:3450–3456. 19064561 10.1158/1055-9965.EPI-08-0311

[27] Govender L, Abel B, Hughes EJ, Scriba TJ, Kagina BM, et al (2010) Higher human CD4 T cell response to novel Mycobacterium tuberculosis latency associated antigens Rv2660 and Rv2659 in latent infection compared with tuberculosis disease. Vaccine 29:51–57. 20974305 10.1016/j.vaccine.2010.10.022 PMC3376751

[28] Hirsch CS, Toossi Z, Vanham G, Johnson JL, Peters P, et al (1999) Apoptosis and T cell hyporesponsiveness in pulmonary tuberculosis. J Infect Dis 179:945–953. 10068591 10.1086/314667

[29] Marin ND, Paris SC, Rojas M, Garcia LF (2013) Functional profile of CD4^+^ and CD8^+^ T cells in latently infected individuals and patients with active TB. Tuberculosis (Edinb) 93:155–166. 23332142 10.1016/j.tube.2012.12.002

[30] Petruccioli E, Petrone L, Vanini V, Sampaolesi A, Gualano G, et al (2013) IFNγ/TNFα specific-–cells and effector memory phenotype associate with active tuberculosis. J Infect 66:475–486. 23462597 10.1016/j.jinf.2013.02.004

[31] Lalvani A, Brookes R, Wilkinson RJ, Malin AS, Pathan AA, et al (1998) Human cytolytic and interferon gamma-secreting CD8^+^ T lymphocytes specific for Mycobacterium uberculosis. Proc Natl Acad Sci USA 95:270–275. 9419365 10.1073/pnas.95.1.270 PMC18198

[32] Scriba TJ, Kalsdorf B, Abrahams DA, Isaacs F, Hofmeister J, et al (2008) Distinct, specific IL–17− and IL–22–producing CD4+ T cell subsets contribute to the human anti-mycobacterial immune response. J Immunol 180:1962–1970. 18209095 10.4049/jimmunol.180.3.1962 PMC2219462

[33] Desvignes L, Ernst JD (2009) Interferon-gamma-responsive nonhematopoietic cells regulate the immune response to Mycobacterium tuberculosis. Immunity 31:974–985. 20064452 10.1016/j.immuni.2009.10.007 PMC2807991

[34] Perreau M, Rozot V, Welles HC, Belluti–Enders F, Vigano S, et al (2013) Lack of Mycobacterium tuberculosis–specific interleukin–17A-producing CD4+ T cells in active disease. Eur J Immunol 43:939–948. 23436562 10.1002/eji.201243090

[35] Hur YG, Gorak-Stolinska P, Ben-Smith A, Lalor MK, Chaguluka S, et al (2013) Combination of cytokine responses indicative of latent TB and active TB in Malawian adults. PLoS One 8:e79742. 24260295 10.1371/journal.pone.0079742 PMC3832606

[36] van Brummelen SE, Bauwens AM, Schlösser NJ, Arend SM (2010) Kinetics of a tuberculosis–specific gamma interferon release assay in military personnel with a positive tuberculin skin test. Clin Vaccine Immunol 17:937–943. 20375241 10.1128/CVI.00005-10 PMC2884429

[37] Felber A, Graninger W (2013) Weakly positive tests and chronologic variation of the QuantiFERON assay: a retrospective appraisal of usefulness. Tuberculosis (Edinb) 93:647–653. 23978524 10.1016/j.tube.2013.07.006

[38] Salaman MR (2013) Persistent negative tuberculin reactors. Tuberculosis (Edinb) 93:688–689. 24074512 10.1016/j.tube.2013.08.007

[39] Tao L, Zalwango S, Chervenak K, Thiel B, Malone LL, et al (2013) Genetic and shared environmental influences on interferon-γ production in response to Mycobacterium tuberculosis antigens in a Ugandan population. Am J Trop Med Hyg 89:169–173. 23629934 10.4269/ajtmh.12-0670 PMC3748477

[40] Jepson A, Fowler A, Banya W, Singh M, Bennett S, et al (2001) Genetic regulation of acquired immune responses to antigens of Mycobacterium tuberculosis: a study of twins in West Africa. Infect Immun 69:3989–3994. 11349068 10.1128/IAI.69.6.3989-3994.2001 PMC98461

[41] Altet–Gómez N, De Souza–Galvao M, Latorre I, Milà C, Jiménez MA, et al (2011) Diagnosing TB infection in children: analysis of discordances using in vitro tests and the tuberculin skin test. Eur Respir J 37:1166–1174. 20729220 10.1183/09031936.00022710

[42] Mancuso JD, Mazurek GH, Tribble D, Olsen C, Aronson NE, et al (2012) Discordance among commercially available diagnostics for latent tuberculosis infection. Am J Respir Crit Care Med 185:427–434. 22161162 10.1164/rccm.201107-1244OC PMC3297098

[43] Gallant CJ, Cobat A, Simkin L, Black GF, Stanley K, et al (2010) Tuberculin skin test and *in vitro* assays provide complementary measures of antimycobacterial immunity in children and adolescents. Chest 137:1071–1077. 20040612 10.1378/chest.09-1852

