# Supplementary material for: Broad Adaptive Immune Responses to M. tuberculosis Antigens Precede TST Conversion in Tuberculosis Exposed Household Contacts in a TB-Endemic Setting
Source: PLoS One. 2014 Dec 30;9(12):e116268. doi: 10.1371/journal.pone.0116268 (PMC4280211; doi:10.1371/journal.pone.0116268)
Supplement: S1 Table — Cytokine/chemokine responses of cases and contacts after 1 day stimulation with ESAT–6/CFP–10 (EC). The geometric mean (GM) levels are shown in pg/ml and the ratio of the geometric mean levels is compared to TST+ contacts. P–values are shown for the unadjusted analysis and after adjustment for household, sex and age. ns = not significant = p>0.05. TST+ = TST positive at baseline; TSTC = TST converters; PTST− = persistently TST negative. (DOCX) [file pone.0116268.s001.docx]

**Table S1: Cytokine/chemokine responses of cases and contacts after 1 day stimulation with ESAT-6/CFP-10**

|  |  |  | **Unadjusted** | | | | **Adjusted for household, sex, age** | | | |
| --- | --- | --- | --- | --- | --- | --- | --- | --- | --- | --- |
| **Analyte** | **Status** | **GM (pg/ml)** | **Ratio GMs** | **p-value vs** | | | **Ratio GMs** | **p-value vs** | | |
|  |  |  |  | **TST+** | **TSTC** | **PTST-** |  | **TST+** | **TSTC** | **PTST-** |
| **IFN-γ** | TST+ | **3.7** | **1** |  |  |  | 1 |  |  |  |
|  | TSTC | **3.5** | **1.0** | ns |  |  | **1.4** | ns |  |  |
|  | PTST- | **0.7** | **0.2** | 0.001 | 0.005 |  | **0.3** | 0.054 | 0.022 |  |
|  | TB Case | **26.9** | **7.3** | 0.001 | 0.002 | 0.000 | **4.5** | 0.047 | ns | 0.001 |
| **IL-2** | TST+ | **1.5** | **1** |  |  |  | **1** |  |  |  |
|  | TSTC | **1.6** | **1.0** | ns |  |  | **1.2** | ns |  |  |
|  | PTST- | **0.3** | **0.2** | 0.000 | 0.002 |  | **0.2** | 0.025 | 0.013 |  |
|  | TB Case | **8.1** | **5.4** | 0.002 | 0.011 | 0.000 | **3.0** | ns | ns | 0.004 |
| **TNF-α** | TST+ | **23.3** | **1** |  |  |  | **1** |  |  |  |
|  | TSTC | **61.9** | **2.7** | ns |  |  | **3.4** | ns |  |  |
|  | PTST- | **27.9** | **1.2** | ns | ns |  | **1.2** | ns | ns |  |
|  | TB Case | **160.4** | **6.9** | 0.001 | ns | 0.004 | **4.4** | 0.022 | ns | ns |
| **MCP-3** | TST+ | **4.0** | **1** |  |  |  | **1** |  |  |  |
|  | TSTC | **2.0** | **0.5** | ns |  |  | **0.5** | 0.054 |  |  |
|  | PTST- | **2.9** | **0.7** | ns | ns |  | **0.8** | ns | ns |  |
|  | TB Case | **14.6** | **3.6** | 0.007 | 0.000 | 0.002 | **2.8** | ns *(0.062)* | 0.003 | 0.041 |
| **IP-10** | TST+ | **1.4** | **1** |  |  |  | **1** |  |  |  |
|  | TSTC | **0.7** | **0.5** | ns |  |  | **0.4** | ns |  |  |
|  | PTST- | **0.9** | **0.6** | ns | ns |  | **0.7** | ns | ns |  |
|  | TB Case | **5.9** | **4.1** | ns *(0.066)* | 0.012 | 0.016 | **3.1** | ns | 0.009 | 0.052 |
| **IL-13** | TST+ | **0.3** | **1** |  |  |  | **1** |  |  |  |
|  | TSTC | **0.4** | **1.3** | ns |  |  | **1.5** | ns |  |  |
|  | PTST- | **0.2** | **0.7** | ns | ns |  | **0.6** | ns | 0.04 |  |
|  | TB Case | **1.2** | **3.6** | 0.006 | ns *(0.055)* | 0.000 | **2.6** | ns | ns | 0.024 |
