# Supplementary material for: Broad Adaptive Immune Responses to M. tuberculosis Antigens Precede TST Conversion in Tuberculosis Exposed Household Contacts in a TB-Endemic Setting
Source: PLoS One. 2014 Dec 30;9(12):e116268. doi: 10.1371/journal.pone.0116268 (PMC4280211; doi:10.1371/journal.pone.0116268)
Supplement: S2 Table — Cytokine/chemokine responses of cases and contacts after 6 day stimulation with ESAT–6/CFP–10. The geometric mean (GM) levels are shown in pg/ml and the ratio of the geometric mean levels is compared to TST+ contacts. P–values are shown for the unadjusted analysis and after adjustment for household, sex and age. ns = not significant. TST+ = TST positive at baseline; TSTC = TST converters; PTST− = persistently TST negative. (DOCX) [file pone.0116268.s002.docx]

**Table S2:** **Cytokine/chemokine responses of cases and contacts after 6 day stimulation with ESAT-6/CFP-10**

|  |  |  | **Unadjusted** | | | | **Adjusted for household, sex, age** | | | |
| --- | --- | --- | --- | --- | --- | --- | --- | --- | --- | --- |
| **Analyte** | **Status** | **GM (pg/ml)** | **Ratio GMs** | **p-value vs** | | | **Ratio GMs** | **p-value vs** | | |
|  |  |  |  | **TST+** | **TSTC** | **PTST-** |  | **TST+** | **TSTC** | **PTST-** |
| **IFN-γ** | TST+ | **119.5** | 1 |  |  |  | 1 |  |  |  |
|  | TSTC | **98.4** | **0.8** | ns |  |  | **1.9** | ns |  |  |
|  | PTST- | **7.0** | **0.1** | 0.000 | 0.003 |  | **0.1** | 0.027 | 0.004 |  |
|  | TB Case | **86.4** | **0.7** | ns | ns | 0.001 | **0.7** | ns | ns | ns |
| **IP-10** | TST+ | **75.6** | **1** |  |  |  | **1** |  |  |  |
|  | TSTC | **138.2** | **1.8** | ns |  |  | **3.2** | ns |  |  |
|  | PTST- | **13.9** | **0.2** | 0.002 | 0.001 |  | **0.3** | ns | 0.004 |  |
|  | TB Case | **330.9** | **4.4** | 0.030 | ns | 0.000 | **2.2** | ns | ns | 0.013 |
| **GMCSF** | TST+ | **45.3** | **1** |  |  |  | **1** |  |  |  |
|  | TSTC | **54.1** | **1.2** | ns |  |  | **1.9** | ns |  |  |
|  | PTST- | **5.1** | **0.1** | 0.000 | 0.001 |  | **0.2** | ns *(0.068)* | 0.005 |  |
|  | TB Case | **70.0** | **1.6** | ns | ns | 0.000 | **1.1** | ns | ns | ns |
| **MIP-1β** | TST+ | **348.7** | **1** |  |  |  | **1** |  |  |  |
|  | TSTC | **463.4** | **1.3** | ns |  |  | **2.1** | ns |  |  |
|  | PTST- | **43.0** | **0.1** | 0.001 | 0.002 |  | **0.2** | 0.046 | 0.007 |  |
|  | TB Case | **278.5** | **0.8** | ns | ns | 0.027 | **0.7** | ns | ns | ns |
| **IL-2RA** | TST+ | **13.6** | **1** |  |  |  | **1** |  |  |  |
|  | TSTC | **18.6** | **1.4** | ns |  |  | **2.3** | ns |  |  |
|  | PTST- | **1.1** | **0.8** | 0.000 | 0.001 |  | **1.7** | 0.033 | 0.003 |  |
|  | TB Case | **11.8** | **0.9** | ns | ns | 0.003 | **0.8** | ns | ns | ns |
| **TNF-α** | TST+ | **53.5** | **1** |  |  |  | **1** |  |  |  |
|  | TSTC | **32.6** | **0.6** | ns |  |  | **0.8** | ns |  |  |
|  | PTST- | **10.0** | **0.2** | 0.002 | ns |  | **0.4** | ns | ns |  |
|  | TB Case | **159.1** | **3.0** | 0.050 | 0.019 | 0.000 | **1.7** | ns | ns | ns |
| **MCP-3** | TST+ | **24.6** | **1** |  |  |  | **1** |  |  |  |
|  | TSTC | **27.7** | **1.1** | ns |  |  | **1.5** | ns |  |  |
|  | PTST- | **5.2** | **0.2** | 0.001 | 0.004 |  | **0.4** | ns *(0.062)* | 0.026 |  |
|  | TB Case | **43.2** | **1.8** | ns | ns | 0.001 | **1.0** | ns | ns | ns |
| **IL-13** | TST+ | **9.1** | **1** |  |  |  | **1** |  |  |  |
|  | TSTC | **7.4** | **0.8** | ns |  |  | **1.0** | ns |  |  |
|  | PTST- | **1.2** | **0.1** | 0.000 | 0.007 |  | **0.2** | 0.027 | 0.037 |  |
|  | TB Case | **10.6** | **1.2** | ns | ns | 0.001 | **1.3** | ns | ns | 0.038 |
| **IL-17** | TST+ | **0.8** | **1** |  |  |  | **1** |  |  |  |
|  | TSTC | **1.3** | **1.5** | ns |  |  | **2.6** | ns |  |  |
|  | PTST- | **0.3** | **0.4** | 0.022 | 0.019 |  | **0.7** | ns | 0.041 |  |
|  | TB Case | **0.7** | **0.8** | ns | ns | ns | **0.8** | ns | ns | ns |
| **IL-10** | TST+ | **2.0** | **1** |  |  |  | **1** |  |  |  |
|  | TSTC | **2.2** | **1.1** | ns |  |  | **1.1** | ns |  |  |
|  | PTST- | **0.6** | **0.3** | 0.005 | 0.024 |  | **0.4** | ns | ns |  |
|  | TB Case | **1.3** | **0.7** | ns | ns | ns | **0.7** | ns | ns | ns |
| **IL-1A** | TST+ | **5.0** | **1** |  |  |  | **1** |  |  |  |
|  | TSTC | **5.7** | **1.1** | ns |  |  | **1.7** | ns |  |  |
|  | PTST- | **1.0** | **0.2** | 0.001 | 0.005 |  | **0.4** | ns | ns *(0.069)* |  |
|  | TB Case | **6.8** | **1.4** | ns | ns | 0.003 | **0.8** | ns | ns | ns |
