# Supplementary material for: Broad Adaptive Immune Responses to M. tuberculosis Antigens Precede TST Conversion in Tuberculosis Exposed Household Contacts in a TB-Endemic Setting
Source: PLoS One. 2014 Dec 30;9(12):e116268. doi: 10.1371/journal.pone.0116268 (PMC4280211; doi:10.1371/journal.pone.0116268)
Supplement: S3 Table — Cytokine/chemokine responses of contacts stratified by TST and ESAT–6/CFP–10 ELISPOT (ECS) results after 1 day stimulation with ESAT–6/CFP-10. The geometric mean (GM) levels are shown in pg/ml and the ratio of the geometric mean levels is compared to TST+ECS− contacts. P-values are shown for the unadjusted analysis and after adjustment for household, sex and age. ns = not significant. ECS+ = positive EC ELISPOT; ECS− = negative EC ELISPOT; TST+ = TST positive at baseline; TSTC = TST converters; PTST− = persistently TST negative. (DOCX) [file pone.0116268.s003.docx]

**Table S3: Cytokine/chemokine responses of contacts stratified by TST and ESAT-6/CFP-10 ELISPOT (ECS) results after 1 day stimulation with ESAT-6/CFP-10**

|  |  |  | **Unadjusted** | | | | | | **Adjusted for household, sex, age** | | | | | |
| --- | --- | --- | --- | --- | --- | --- | --- | --- | --- | --- | --- | --- | --- | --- |
| **Analyte** | **Status** | **GM**  **(pg/ml)** | **Ratio**  **GMs** | **p-value vs** | | | | | **Ratio**  **GMs** | **p-value vs** | | | | |
|  |  |  |  | **TST+**  **ECS-** | **TST+**  **ECS+** | **TSTC**  **ECS-** | **TSTC**  **ECS+** | **PTST-ECS-** |  | **TST+**  **ECS-** | **TST+**  **ECS+** | **TSTC**  **ECS-** | **TSTC**  **ECS+** | **PTST-ECS-** |
| **IFN-γ** | TST+ECS- | **0.9** | **1** |  |  |  |  |  | **1** |  |  |  |  |  |
|  | TST+ECS+ | **16.3** | **18.8** | 0.000 |  |  |  |  | **10.0** | 0.003 |  |  |  |  |
|  | TSTCECS- | **1.8** | **2.1** | ns | 0.002 |  |  |  | **1.4** | ns | 0.023 |  |  |  |
|  | TSTCECS+ | **12.7** | **14.6** | 0.001 | ns | 0.028 |  |  | **20.3** | 0.000 | ns | 0.005 |  |  |
|  | PTST-ECS- | **0.7** | **0.8** | ns | 0.000 | ns | 0.000 |  | **0.7** | ns | 0.000 | ns | 0.000 |  |
|  | PTST-ECS+ | **1.1** | **1.2** | ns | 0.025 | ns | ns *(0.062)* | ns | **1.3** | ns | ns | ns | 0.020 | ns |
| **IL-2** | TST+ECS- | **0.2** | **1** |  |  |  |  |  | **1** |  |  |  |  |  |
|  | TST+ECS+ | **11.1** | **51.4** | 0.000 |  |  |  |  | **40.2** | 0.000 |  |  |  |  |
|  | TSTCECS- | **0.7** | **3.3** | ns | 0.000 |  |  |  | **2.8** | ns | 0.001 |  |  |  |
|  | TSTCECS+ | **7.0** | **32.3** | 0.000 | ns | 0.010 |  |  | **37.5** | 0.000 | ns | 0.007 |  |  |
|  | PTST-ECS- | **0.2** | **0.9** | ns | 0.000 | 0.045 | 0.000 |  | **0.7** | ns | 0.000 | ns *(0.069*) | 0.000 |  |
|  | PTST-ECS+ | **2.0** | **9.1** | 0.022 | ns | ns | ns | 0.015 | **12.9** | 0.002 | ns | ns | ns | 0.000 |
| **IP-10** | TST+ECS- | **0.7** | **1** |  |  |  |  |  | **1** |  |  |  |  |  |
|  | TST+ECS+ | **2.9** | **3.9** | 0.029 |  |  |  |  | **2.3** | ns |  |  |  |  |
|  | TSTCECS- | **1.1** | **1.5** | ns | ns |  |  |  | **0.8** | ns | ns |  |  |  |
|  | TSTCECS+ | **0.3** | **0.4** | 0.012 | 0.000 | ns *(0.066)* |  |  | **0.4** | ns | ns | ns |  |  |
|  | PTST-ECS- | **0.8** | **1.1** | ns | 0.048 | ns | 0.01 |  | **0.9** | ns | ns | ns | ns |  |
|  | PTST-ECS+ | **1.4** | **1.9** | ns | ns | ns | ns | ns | **1.4** | ns | ns | ns | ns | ns |
| **MCP-3** | TST+ECS- | **2.5** | **1** |  |  |  |  |  | **1** |  |  |  |  |  |
|  | TST+ECS+ | **6.5** | **2.6** | 0.029 |  |  |  |  | **2.2** | ns |  |  |  |  |
|  | TSTCECS- | **3.0** | **1.2** | ns | ns |  |  |  | **0.7** | ns | ns |  |  |  |
|  | TSTCECS+ | **0.9** | **0.4** | 0.001 | 0.000 | 0.040 |  |  | **0.4** | ns | ns | ns |  |  |
|  | PTST-ECS- | **3.0** | **1.2** | ns | ns | ns | 0.002 |  | **1.1** | ns | ns | ns | ns |  |
|  | PTST-ECS+ | **2.5** | **1.0** | ns | ns | ns | ns | ns | **0.6** | ns | ns | ns | ns | ns |
| **IL-17** | TST+ECS- | **0.1** | **1** |  |  |  |  |  | **1** |  |  |  |  |  |
|  | TST+ECS+ | **0.1** | **1.1** | ns |  |  |  |  | **1.1** | ns |  |  |  |  |
|  | TSTCECS- | **0.1** | **0.8** | ns | ns |  |  |  | **1.2** | ns | ns |  |  |  |
|  | TSTCECS+ | **0.1** | **0.7** | 0.024 | 0.018 | ns |  |  | **0.7** | ns | ns | ns |  |  |
|  | PTST-ECS- | **0.1** | **0.9** | ns | ns | ns | 0.023 |  | **1.1** | ns | ns | ns | ns |  |
|  | PTST-ECS+ | **0.2** | **0.2** | ns | ns | ns | ns | ns | **2.0** | ns | ns | ns | ns | ns |
