# Supplementary material for: Broad Adaptive Immune Responses to M. tuberculosis Antigens Precede TST Conversion in Tuberculosis Exposed Household Contacts in a TB-Endemic Setting
Source: PLoS One. 2014 Dec 30;9(12):e116268. doi: 10.1371/journal.pone.0116268 (PMC4280211; doi:10.1371/journal.pone.0116268)
Supplement: S4 Table — Cytokine/chemokine responses of contacts stratified by TST and ESAT-6/CFP-10 ELISPOT (ECS) results after 6 day stimulation with ESAT–6/CFP–10. The geometric mean (GM) levels are shown in pg/ml and the ratio of the geometric mean levels is compared to TST+ECS− contacts. P–values are shown for the unadjusted analysis and after adjustment for household, sex and age. ns = not significant; ECS+ = positive EC ELISPOT; ECS− = negative EC ELISPOT; TST+ = TST positive at baseline; TSTC = TST converters; PTST− = persistently TST negative. (DOCX) [file pone.0116268.s004.docx]

**Table S4: Cytokine/chemokine responses of contacts stratified by TST and ESAT-6/CFP-10 ELISPOT (ECS) results after 6 day stimulation with ESAT-6/CFP-10**

|  |  |  |  | **Unadjusted** | | | | |  | **Adjusted for household, sex, age** | | | | |
| --- | --- | --- | --- | --- | --- | --- | --- | --- | --- | --- | --- | --- | --- | --- |
| **Analyte** | **Status** | **GM**  **(pg/ml)** | **Ratio**  **GMs** | **p-value vs** | | | | | **Ratio**  **GMs** | **p-value** | | | | |
|  |  |  |  | **TST+**  **ECS-** | **TST+**  **ECS+** | **TSTC**  **ECS-** | **TSTC**  **ECS+** | **PTST-ECS-** |  | **TST+**  **ECS-** | **TST+**  **ECS+** | **TSTC**  **ECS-** | **TSTC**  **ECS+** | **PTST-ECS-** |
| **IFN-γ** | TST+ECS- | **12.4** | **1** |  |  |  |  |  | **1** |  |  |  |  |  |
|  | TST+ECS+ | **1243.6** | **100.7** | 0.000 |  |  |  |  | **64.2** | 0.000 |  |  |  |  |
|  | TSTCECS- | **30.3** | **2.5** | ns | 0.001 |  |  |  | **5.3** | ns | 0.024 |  |  |  |
|  | TSTCECS+ | **908.7** | **73.6** | 0.000 | ns | 0.004 |  |  | **54.2** | 0.000 | ns | ns *(0.061)* |  |  |
|  | PTST-ECS- | **4.0** | **0.3** | ns | 0.000 | ns *(0.060)* | 0.000 |  | **0.6** | ns | 0.000 | ns *(0.058)* | 0.000 |  |
|  | PTST-ECS+ | **313.5** | **25.4** | 0.000 | 0.030 | 0.034 | ns | 0.000 | **80.5** | 0.001 | ns | ns | ns | 0.000 |
| **IP-10** | TST+ECS- | **12.5** | **1** |  |  |  |  |  | **1** |  |  |  |  |  |
|  | TST+ECS+ | **485.9** | **38.9** | 0.000 |  |  |  |  | **16.5** | 0.000 |  |  |  |  |
|  | TSTCECS- | **42.4** | **3.4** | ns | 0.004 |  |  |  | **2.6** | ns | ns *(0.055)* |  |  |  |
|  | TSTCECS+ | **1287.6** | **103.2** | 0.000 | ns | 0.000 |  |  | **82.3** | 0.000 | 0.037 | 0.001 |  |  |
|  | PTST-ECS- | **7.9** | **0.6** | ns | 0.000 | 0.033 | 0.000 |  | **0.6** | ns | 0.000 | ns | 0.000 |  |
|  | PTST-ECS+ | **666.2** | **53.4** | 0.000 | ns | 0.001 | ns | 0.000 | **49.8** | 0.001 | ns | 0.034 | ns | 0.000 |
| **GMCSF** | TST+ECS- | **6.2** | **1** |  |  |  |  |  | **1** |  |  |  |  |  |
|  | TST+ECS+ | **353.0** | **57.0** | 0.000 |  |  |  |  | **31.2** | 0.000 |  |  |  |  |
|  | TSTCECS- | **19.1** | **3.1** | ns | 0.000 |  |  |  | **5.7** | ns | 0.051 |  |  |  |
|  | TSTCECS+ | **384.2** | **62.0** | 0.000 | ns | 0.000 |  |  | **29.8** | 0.000 | ns | ns  *(0.063)* |  |  |
|  | PTST-ECS- | **3.5** | **0.6** | ns | 0.000 | 0.024 | 0.000 |  | **0.9** | ns | 0.000 | 0.046 | 0.000 |  |
|  | PTST-ECS+ | **60.4** | **9.7** | ns | ns | ns | ns | ns  *(0.067*) | **11.8** | ns *(0.061)* | ns | ns | ns | 0.034 |
| **MIP-1β** | TST+ECS- | **54.7** | **1** |  |  |  |  |  | **1** |  |  |  |  |  |
|  | TST+ECS+ | **2361.6** | **43.2** | 0.000 |  |  |  |  | **27.6** | 0.000 |  |  |  |  |
|  | TSTCECS- | **240.0** | **4.4** | ns | 0.005 |  |  |  | **8.5** | 0.051 | ns |  |  |  |
|  | TSTCECS+ | **1605.5** | **29.4** | 0.002 | ns | ns |  |  | **23.4** | 0.009 | ns | ns |  |  |
|  | PTST-ECS- | **30.9** | **0.6** | ns | 0.000 | 0.016 | 0.000 |  | **0.8** | ns | 0.000 | 0.015 | 0.004 |  |
|  | PTST-ECS+ | **413.4** | **7.6** | ns | ns | ns | ns | ns | **17.3** | 0.015 | ns | ns | ns | 0.004 |
| **TNF-α** | TST+ECS- | **11.1** | **1** |  |  |  |  |  | **1** |  |  |  |  |  |
|  | TST+ECS+ | **270.4** | **24.3** | 0.000 |  |  |  |  | **14.3** | 0.001 |  |  |  |  |
|  | TSTCECS- | **10.6** | **1.0** | ns | 0.000 |  |  |  | **1.3** | ns | 0.006 |  |  |  |
|  | TSTCECS+ | **270.0** | **24.2** | 0.000 | ns | 0.000 |  |  | **13.1** | 0.001 | ns | 0.018 |  |  |
|  | PTST-ECS- | **7.8** | **0.7** | ns | 0.000 | ns | 0.000 |  | **1.0** | ns | 0.002 | ns | 0.002 |  |
|  | PTST-ECS+ | **56.2** | **5.0** | ns | ns | ns | ns | ns | **6.6** | ns | ns | ns | ns | ns |
| **MCP-3** | TST+ECS- | **4.5** | **1** |  |  |  |  |  | **1** |  |  |  |  |  |
|  | TST+ECS+ | **141.1** | **31.1** | 0.000 |  |  |  |  | **19.2** | 0.000 |  |  |  |  |
|  | TSTCECS- | **10.8** | **2.4** | ns | 0.000 |  |  |  | **2.4** | ns | 0.006 |  |  |  |
|  | TSTCECS+ | **166.0** | **36.5** | 0.000 | ns | 0.001 |  |  | **19.9** | 0.000 | ns | 0.022 |  |  |
|  | PTST-ECS- | **4.3** | **0.9** | ns | 0.000 | ns | 0.000 |  | **1.2** | ns | 0.000 | ns | 0.000 |  |
|  | PTST-ECS+ | **21.1** | **4.6** | ns | ns | ns | ns | ns | **3.7** | ns | ns | ns | ns | ns |
| **IL-2RA** | TST+ECS- | **1.6** | **1** |  |  |  |  |  | **1** |  |  |  |  |  |
|  | TST+ECS+ | **122.9** | **76.4** | 0.000 |  |  |  |  | **37.7** | 0.000 |  |  |  |  |
|  | TSTCECS- | **5.5** | **3.4** | ns | 0.003 |  |  |  | **4.8** | ns | ns |  |  |  |
|  | TSTCECS+ | **188.9** | **117.4** | 0.000 | ns | 0.000 |  |  | **46.8** | 0.000 | ns | ns |  |  |
|  | PTST-ECS- | **0.7** | **0.5** | ns | 0.000 | 0.041 | 0.000 |  | **0.6** | ns | 0.000 | ns *(0.066)* | 0.000 |  |
|  | PTST-ECS+ | **22.8** | **14.2** | ns *(0.058)* | ns | ns | ns | 0.011 | **35.3** | 0.014 | ns | ns | ns | 0.005 |
| **IL-13** | TST+ECS- | **2.1** | **1** |  |  |  |  |  | **1** |  |  |  |  |  |
|  | TST+ECS+ | **40.9** | **19.2** | 0.000 |  |  |  |  | **10.2** | 0.006 |  |  |  |  |
|  | TSTCECS- | **4.2** | **2.0** | ns | 0.003 |  |  |  | **2.1** | ns | ns |  |  |  |
|  | TSTCECS+ | **21.8** | **10.3** | 0.027 | ns | ns |  |  | **3.9** | ns | ns | ns |  |  |
|  | PTST-ECS- | **0.8** | **0.4** | ns | 0.000 | 0.018 | 0.001 |  | **0.4** | ns | 0.000 | 0.045 | 0.030 |  |
|  | PTST-ECS+ | **28.5** | **13.4** | ns *(0.063)* | ns | ns | ns | 0.008 | **15.4** | ns | ns | ns | ns | 0.022 |
| **IL-17** | TST+ECS- | **0.3** | **1** |  |  |  |  |  | **1** |  |  |  |  |  |
|  | TST+ECS+ | **2.6** | **9.1** | 0.000 |  |  |  |  | **7.0** | 0.004 |  |  |  |  |
|  | TSTCECS- | **0.4** | **1.3** | ns | 0.007 |  |  |  | **2.4** | ns | ns |  |  |  |
|  | TSTCECS+ | **13.2** | **46.9** | 0.000 | 0.003 | 0.000 |  |  | **36.2** | 0.000 | 0.056 | 0.007 |  |  |
|  | PTST-ECS- | **0.2** | **0.8** | ns | 0.000 | ns | 0.000 |  | **1.5** | ns | 0.022 | ns | 0.000 |  |
|  | PTST-ECS+ | **1.7** | **6.1** | ns | ns | ns | ns | ns | **7.7** | ns *(0.067)* | ns | ns | ns | ns |
| **IL-10** | TST+ECS- | **0.6** | **1** |  |  |  |  |  | **1** |  |  |  |  |  |
|  | TST+ECS+ | **6.7** | **10.6** | 0.000 |  |  |  |  | **7.3** | 0.010 |  |  |  |  |
|  | TSTCECS- | **0.8** | **1.2** | ns | 0.004 |  |  |  | **1.3** | ns | 0.047 |  |  |  |
|  | TSTCECS+ | **15.5** | **24.4** | 0.000 | ns | 0.000 |  |  | **11.7** | 0.001 | ns | 0.019 |  |  |
|  | PTST-ECS- | **0.4** | **0.7** | ns | 0.000 | ns | 0.000 |  | **0.9** | ns | 0.007 | ns | 0.000 |  |
|  | PTST-ECS+ | **2.7** | **4.2** | ns | ns | ns | ns | ns | **4.4** | ns | ns | ns | ns | ns |
| **IL-1A** | TST+ECS- | **1.69** | **1** |  |  |  |  |  | **1** |  |  |  |  |  |
|  | TST+ECS+ | **15.4** | **9.1** | 0.000 |  |  |  |  | **6.7** | 0.007 |  |  |  |  |
|  | TSTCECS- | **3.4** | **2.0** | ns | 0.036 |  |  |  | **2.0** | ns | ns |  |  |  |
|  | TSTCECS+ | **15.2** | **9.0** | 0.005 | ns | ns |  |  | **11.8** | 0.005 | ns | ns |  |  |
|  | PTST-ECS- | **0.8** | **0.5** | ns | 0.000 | 0.052 | 0.000 |  | **1.0** | ns | 0.012 | ns | 0.006 |  |
|  | PTST-ECS+ | **5.3** | **3.2** | ns | ns | ns | ns | ns | **5.2** | 0.046 | ns | ns | ns | 0.048 |
